# 2-Anilino-3-(2-hy­droxy­prop­yl)-4-methyl-1,3-thia­zol-3-ium chloride

**DOI:** 10.1107/S1600536812023197

**Published:** 2012-05-26

**Authors:** Shaaban K. Mohamed, Mehmet Akkurt, Muhammad N. Tahir, Antar A. Abdelhamid, Ali N. Khalilov

**Affiliations:** a Chemistry and Environmental Division, Manchester Metropolitan University, Manchester M1 5GD, England; b Department of Physics, Faculty of Sciences, Erciyes University, 38039 Kayseri, Turkey; c University of Sargodha, Department of Physics, Sargodha, Pakistan; d Department of Organic Chemistry, Baku State University, Baku, Azerbaijan

## Abstract

In the title compound, C_13_H_17_N_2_OS^+^·Cl^−^, the thia­zolium ring mean plane makes a dihedral angle of 55.46 (9)° with the benzene ring. In the propanol group, the N—C—C—C and N—C—C—O torsion angles are 172.58 (15) and 52.9 (2)°, respectively, and the S—C—C—C torsion angle is 178.99 (18)°. In the crystal, mol­ecules are linked by O—H⋯Cl and N—H⋯Cl hydrogen bonds, forming zigzag chains along [001]. There is also a C—H⋯Cl inter­action present.

## Related literature

The title compound was prepared as part of an ongoing investigation into the synthesis and biological properties of thia­zole compounds: see; Abdel-Wahab *et al.* (2009 ▶); Baia *et al.* (2008 ▶); Lesyk *et al.* (2007 ▶); Mohamed *et al.* (2012*a* ▶,*b* ▶); Potikha *et al.* (2008 ▶); Shiradkar *et al.* (2007 ▶); Soliman *et al.* (2012 ▶); Wu & Yang (2007 ▶). For related structures, see: Lynch & McClenaghan (2004 ▶); Liu *et al.* (2011 ▶); Wang (2011 ▶).
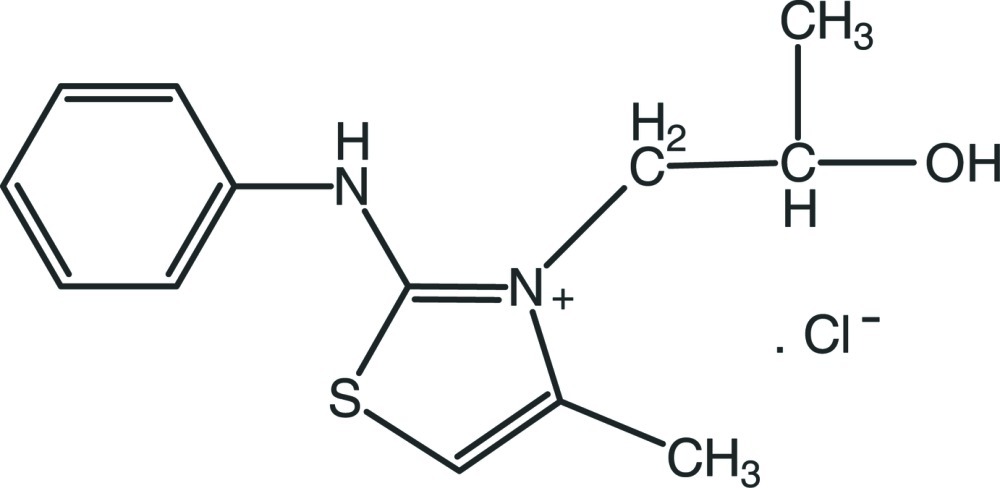


## Experimental

### 

#### Crystal data

C_13_H_17_N_2_OS^+^·Cl^−^
*M* *_r_* = 284.81
Monoclinic, 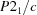

*a* = 11.7570 (4) Å
*b* = 12.2477 (4) Å
*c* = 10.2954 (3) Å
β = 106.532 (1)°
*V* = 1421.21 (8) Å^3^
*Z* = 4
Mo *K*α radiation
μ = 0.41 mm^−1^
*T* = 296 K
0.35 × 0.22 × 0.20 mm

#### Data collection

Bruker Kappa APEXII CCD diffractometer
Absorption correction: multi-scan (*SADABS*; Bruker, 2005 ▶) *T*_min_ = 0.898, *T*_max_ = 0.922
10535 measured reflections
2641 independent reflections
2172 reflections with *I* > 2σ(*I*)
*R*_int_ = 0.024

#### Refinement

*R*[*F* ^2^ > 2σ(*F* ^2^)] = 0.032
*wR*(*F* ^2^) = 0.084
*S* = 1.03
2641 reflections
166 parameters
H-atom parameters constrained
Δρ_max_ = 0.17 e Å^−3^
Δρ_min_ = −0.18 e Å^−3^

### 

Data collection: *APEX2* (Bruker, 2007 ▶); cell refinement: *SAINT* (Bruker, 2007 ▶); data reduction: *SAINT*; program(s) used to solve structure: *SHELXS97* (Sheldrick, 2008 ▶); program(s) used to refine structure: *SHELXL97* (Sheldrick, 2008 ▶); molecular graphics: *ORTEP-3 for Windows* (Farrugia, 1997 ▶) and *PLATON* (Spek, 2009 ▶); software used to prepare material for publication: *WinGX* (Farrugia, 1999 ▶) and *PLATON*.

## Supplementary Material

Crystal structure: contains datablock(s) global, I. DOI: 10.1107/S1600536812023197/su2431sup1.cif

Structure factors: contains datablock(s) I. DOI: 10.1107/S1600536812023197/su2431Isup2.hkl

Supplementary material file. DOI: 10.1107/S1600536812023197/su2431Isup3.cml

Additional supplementary materials:  crystallographic information; 3D view; checkCIF report

## Figures and Tables

**Table 1:** Hydrogen-bond geometry (Å, °)

*D*—H⋯*A*	*D*—H	H⋯*A*	*D*⋯*A*	*D*—H⋯*A*
O1—H1*A*⋯Cl1	0.82	2.36	3.1681 (16)	169
N1—H1⋯Cl1^i^	0.86	2.34	3.1675 (14)	163
C11—H11*A*⋯Cl1^i^	0.97	2.81	3.6440 (19)	144

Symmetry code: (i) 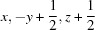.

**Fig. 1.**
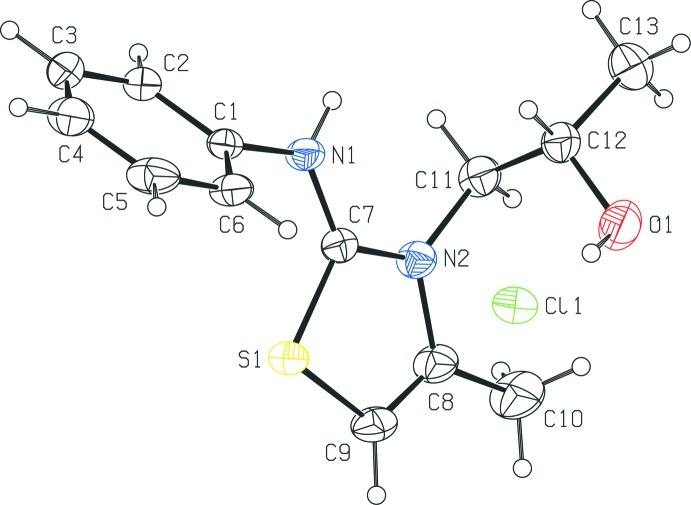
The molecular structure and atom-numbering scheme for the title compound. Displacement ellipsoids are drawn at the 30% probability level.

**Fig. 2.**
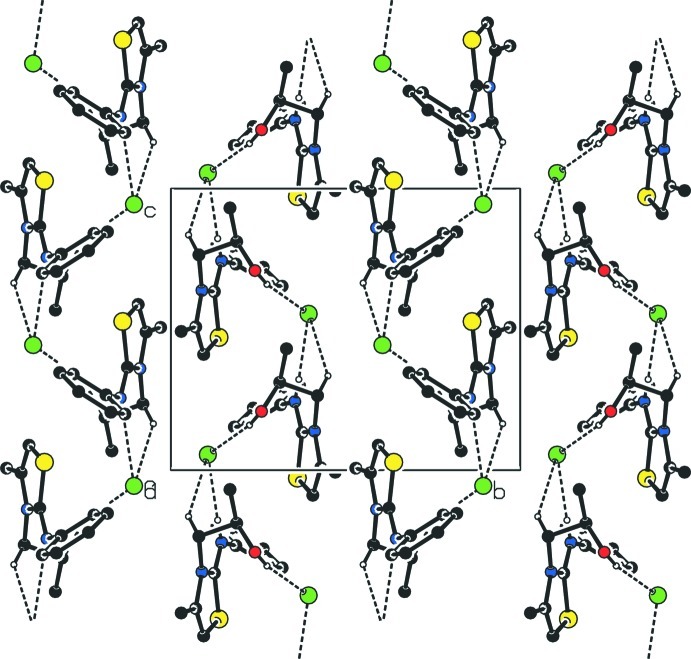
The crystal packing of the title compound, viewing along the a axis. H atoms not involved in hydrogen bonds (dashed lines) have been omitted for clarity.

## supplementary crystallographic information

### Comment

Natural compounds such as, bistratamide H, archazolid A & B, siomycin A, didmolamide A, scleritodermin A, *etc*. (Wu & Yang, 2007) and thiamine (vitamin B1) (Baia *et al.*, 2008), were found to have a thiazol ring system. In addition, thiazole compounds have been reported to exhibit different pharmaceutical properties, for example antibacterial, antifungal (Abdel-Wahab *et al.*, 2009), antitubercular (Shiradkar *et al.*, 2007), anticancer (Lesyk *et al.*, 2007). These compounds have been synthesized using different methods (Potikha *et al.*, 2008). Further to our interest of bioactive compounds (Mohamed *et al.*, 2012**a*,b*; Soliman *et al.*, 2012) we were interested in synthesizing new amino-thiazole derivatives *via* a one pot reaction protocol. We report herein on the synthesis and crystal structure of the title compound.

In the title compound, (Fig. 1), the dihedral angle between the thiazole ring (S1/N2/C7–C9) and the benzene ring (C1–C6) is 55.46 (9)°. The thiazolium ring is essentialy co-planar with the methyl group which is attached to it, with torsion angle S1—C9—C8—C10 being 178.99 (18)°. In the propanol group, torsion angles N2–C11–C12–C13 and N2–C11–C12–O1 are 172.58 (15) and 52.9 (2)°, respectively. Bond lengths and angles have normal values and are comparable to those reported for similar structures (Lynch & McClenaghan, 2004; Liu *et al.*, 2011; Wang, 2011).

In the crystal, molecules are linked by O-H···Cl, N—H···Cl and C—H···Cl hydrogen bonds, into infinite zigzag chains propagating along the [001] direction (Table 1, Fig. 2).

### Experimental

A mixture of 75 mg (1 mmol) 1-aminopropan-2-ol, 135 mg (1 mmol) phenyl isothiocyanate and 93 mg (1 mmol) 1-chloropropan-2-one in 50 ml ethanol was refluxed at 351 K. The reaction was monitored by TLC until completion after four hours then cooled to room temperature. The resulting solid was filtered off, dried under vacuum and recrystallized from ethanol to afford colourless crystals suitable for X-ray analysis [Yield 79%; M.p. 419 K].

### Refinement

The H atoms were located in a difference Fourier map. In the final cycles of refinement they were included in calculated poitions and refined using a riding model: N—H = 0.86 Å and C—H = 0.93 Å (aromatic), 0.96 Å (methyl), 0.97 Å (methylene) and 0.98 Å (methine), with *U*_iso_(H) = 1.5*U*_eq_(C) for methyl groups and = 1.2*U*_eq_(C,*N*) for other H atoms.

### Crystal data

**Table:**

C_13_H_17_N_2_OS^+^·Cl^−^	*F*(000) = 600
*M**_r_* = 284.81	*D*_x_ = 1.331 Mg m^−^^3^
Monoclinic, *P*2_1_/*c*	Mo *K*α radiation, λ = 0.71073 Å
Hall symbol: -P 2ybc	Cell parameters from 447 reflections
*a* = 11.7570 (4) Å	θ = 3.5–21.2°
*b* = 12.2477 (4) Å	µ = 0.41 mm^−^^1^
*c* = 10.2954 (3) Å	*T* = 296 K
β = 106.532 (1)°	Rod, colourless
*V* = 1421.21 (8) Å^3^	0.35 × 0.22 × 0.20 mm
*Z* = 4	

### Data collection

**Table:**

Bruker Kappa APEXII CCD diffractometer	2641 independent reflections
Radiation source: fine-focus sealed tube	2172 reflections with *I* > 2σ(*I*)
Graphite monochromator	*R*_int_ = 0.024
Detector resolution: 0.81 pixels mm^-1^	θ_max_ = 25.5°, θ_min_ = 2.5°
ω scans	*h* = −14→11
Absorption correction: multi-scan (*SADABS*; Bruker, 2005)	*k* = −14→14
*T*_min_ = 0.898, *T*_max_ = 0.922	*l* = −12→12
10535 measured reflections	

### Refinement

**Table:**

Refinement on *F*^2^	Primary atom site location: structure-invariant direct methods
Least-squares matrix: full	Secondary atom site location: difference Fourier map
*R*[*F*^2^ > 2σ(*F*^2^)] = 0.032	Hydrogen site location: inferred from neighbouring sites
*wR*(*F*^2^) = 0.084	H-atom parameters constrained
*S* = 1.03	*w* = 1/[σ^2^(*F*_o_^2^) + (0.0393*P*)^2^ + 0.3249*P*] where *P* = (*F*_o_^2^ + 2*F*_c_^2^)/3
2641 reflections	(Δ/σ)_max_ < 0.001
166 parameters	Δρ_max_ = 0.17 e Å^−^^3^
0 restraints	Δρ_min_ = −0.18 e Å^−^^3^

### Special details

**Table:**

Geometry. Bond distances, angles *etc*. have been calculated using the rounded fractional coordinates. All su's are estimated from the variances of the (full) variance-covariance matrix. The cell e.s.d.'s are taken into account in the estimation of distances, angles and torsion angles
Refinement. Refinement on *F*^2^ for ALL reflections except those flagged by the user for potential systematic errors. Weighted *R*-factors *wR* and all goodnesses of fit *S* are based on *F*^2^, conventional *R*-factors *R* are based on *F*, with *F* set to zero for negative *F*^2^. The observed criterion of *F*^2^ > σ(*F*^2^) is used only for calculating -*R*-factor-obs *etc*. and is not relevant to the choice of reflections for refinement. *R*-factors based on *F*^2^ are statistically about twice as large as those based on *F*, and *R*-factors based on ALL data will be even larger.

### Fractional atomic coordinates and isotropic or equivalent isotropic displacement parameters (Å^2^)

**Table:**

	*x*	*y*	*z*	*U*_iso_*/*U*_eq_	
S1	0.53335 (4)	0.36209 (4)	−0.02819 (4)	0.0436 (2)	
O1	0.92376 (12)	0.25939 (14)	0.20894 (15)	0.0722 (6)	
N1	0.58597 (12)	0.35477 (12)	0.24335 (13)	0.0424 (4)	
N2	0.73113 (12)	0.41117 (12)	0.13880 (14)	0.0447 (5)	
C1	0.47013 (14)	0.31414 (14)	0.23234 (15)	0.0382 (5)	
C2	0.40120 (15)	0.36823 (15)	0.30052 (17)	0.0455 (6)	
C3	0.29021 (17)	0.3281 (2)	0.29410 (19)	0.0586 (7)	
C4	0.24821 (17)	0.2359 (2)	0.22085 (19)	0.0673 (8)	
C5	0.31707 (18)	0.18174 (19)	0.15336 (18)	0.0612 (7)	
C6	0.42873 (16)	0.22021 (15)	0.15893 (16)	0.0471 (6)	
C7	0.62227 (15)	0.37561 (13)	0.13445 (16)	0.0384 (5)	
C8	0.74511 (17)	0.42898 (16)	0.00903 (19)	0.0522 (7)	
C9	0.64774 (18)	0.40625 (15)	−0.08878 (19)	0.0527 (7)	
C10	0.8594 (2)	0.4705 (2)	−0.0071 (2)	0.0812 (9)	
C11	0.82819 (15)	0.41979 (16)	0.26481 (18)	0.0509 (6)	
C12	0.88729 (16)	0.31168 (18)	0.31206 (19)	0.0544 (7)	
C13	0.99588 (18)	0.3309 (2)	0.4314 (2)	0.0775 (9)	
Cl1	0.71127 (4)	0.10568 (4)	0.05541 (4)	0.0553 (2)	
H1	0.63390	0.36620	0.32250	0.0510*	
H1A	0.86790	0.22510	0.16010	0.1080*	
H2	0.42940	0.43100	0.35010	0.0550*	
H3	0.24340	0.36390	0.33990	0.0700*	
H4	0.17300	0.20970	0.21670	0.0810*	
H5	0.28820	0.11910	0.10390	0.0730*	
H6	0.47560	0.18360	0.11400	0.0560*	
H9	0.64160	0.41330	−0.18050	0.0630*	
H10A	0.85460	0.47390	−0.10170	0.1220*	
H10B	0.92260	0.42230	0.03820	0.1220*	
H10C	0.87460	0.54220	0.03170	0.1220*	
H11A	0.79720	0.44960	0.33520	0.0610*	
H11B	0.88720	0.47040	0.25100	0.0610*	
H12	0.83160	0.26440	0.34050	0.0650*	
H13A	1.05130	0.37610	0.40350	0.1160*	
H13B	1.03240	0.26220	0.46350	0.1160*	
H13C	0.97260	0.36660	0.50290	0.1160*	

### Atomic displacement parameters (Å^2^)

**Table:**

	*U*^11^	*U*^22^	*U*^33^	*U*^12^	*U*^13^	*U*^23^
S1	0.0486 (3)	0.0486 (3)	0.0332 (2)	−0.0003 (2)	0.0112 (2)	0.0015 (2)
O1	0.0544 (8)	0.0909 (12)	0.0737 (10)	0.0018 (8)	0.0221 (8)	−0.0187 (8)
N1	0.0389 (7)	0.0577 (9)	0.0305 (7)	−0.0068 (7)	0.0095 (6)	−0.0046 (6)
N2	0.0429 (8)	0.0501 (9)	0.0424 (8)	−0.0073 (7)	0.0145 (6)	0.0013 (6)
C1	0.0368 (9)	0.0496 (10)	0.0277 (8)	−0.0008 (8)	0.0083 (7)	0.0037 (7)
C2	0.0466 (10)	0.0562 (11)	0.0339 (9)	0.0062 (8)	0.0120 (8)	0.0041 (8)
C3	0.0417 (10)	0.0936 (16)	0.0433 (11)	0.0091 (11)	0.0167 (9)	0.0074 (10)
C4	0.0415 (11)	0.1137 (19)	0.0455 (11)	−0.0179 (12)	0.0106 (9)	0.0083 (12)
C5	0.0641 (13)	0.0781 (15)	0.0387 (10)	−0.0288 (11)	0.0103 (9)	−0.0025 (9)
C6	0.0539 (11)	0.0570 (11)	0.0318 (9)	−0.0069 (9)	0.0146 (8)	−0.0020 (8)
C7	0.0415 (9)	0.0381 (9)	0.0364 (9)	−0.0001 (7)	0.0123 (7)	−0.0007 (7)
C8	0.0578 (11)	0.0555 (12)	0.0491 (11)	−0.0065 (9)	0.0245 (10)	0.0092 (9)
C9	0.0665 (12)	0.0551 (12)	0.0417 (10)	−0.0017 (9)	0.0239 (10)	0.0093 (8)
C10	0.0748 (15)	0.1027 (19)	0.0776 (15)	−0.0227 (14)	0.0402 (13)	0.0156 (14)
C11	0.0423 (10)	0.0619 (12)	0.0494 (11)	−0.0140 (9)	0.0144 (8)	−0.0084 (9)
C12	0.0401 (10)	0.0747 (14)	0.0485 (11)	−0.0024 (9)	0.0127 (8)	−0.0044 (9)
C13	0.0479 (12)	0.117 (2)	0.0612 (13)	−0.0011 (13)	0.0050 (10)	−0.0043 (13)
Cl1	0.0614 (3)	0.0629 (3)	0.0409 (3)	0.0018 (2)	0.0135 (2)	0.0051 (2)

### Geometric parameters (Å, º)

**Table:**

S1—C7	1.7111 (17)	C11—C12	1.510 (3)
S1—C9	1.723 (2)	C12—C13	1.517 (3)
O1—C12	1.407 (2)	C2—H2	0.9300
O1—H1A	0.8200	C3—H3	0.9300
N1—C7	1.333 (2)	C4—H4	0.9300
N1—C1	1.424 (2)	C5—H5	0.9300
N2—C8	1.409 (2)	C6—H6	0.9300
N2—C11	1.468 (2)	C9—H9	0.9300
N2—C7	1.341 (2)	C10—H10A	0.9600
N1—H1	0.8600	C10—H10B	0.9600
C1—C2	1.383 (2)	C10—H10C	0.9600
C1—C6	1.387 (2)	C11—H11A	0.9700
C2—C3	1.378 (3)	C11—H11B	0.9700
C3—C4	1.370 (3)	C12—H12	0.9800
C4—C5	1.378 (3)	C13—H13A	0.9600
C5—C6	1.381 (3)	C13—H13B	0.9600
C8—C9	1.321 (3)	C13—H13C	0.9600
C8—C10	1.490 (3)		
			
C7—S1—C9	90.08 (9)	C4—C3—H3	120.00
C12—O1—H1A	109.00	C3—C4—H4	120.00
C1—N1—C7	121.86 (14)	C5—C4—H4	120.00
C7—N2—C11	123.21 (14)	C4—C5—H5	120.00
C8—N2—C11	123.78 (15)	C6—C5—H5	120.00
C7—N2—C8	112.74 (14)	C1—C6—H6	120.00
C7—N1—H1	119.00	C5—C6—H6	120.00
C1—N1—H1	119.00	S1—C9—H9	124.00
N1—C1—C2	118.53 (15)	C8—C9—H9	124.00
N1—C1—C6	120.85 (15)	C8—C10—H10A	109.00
C2—C1—C6	120.58 (16)	C8—C10—H10B	110.00
C1—C2—C3	119.24 (17)	C8—C10—H10C	109.00
C2—C3—C4	120.57 (19)	H10A—C10—H10B	109.00
C3—C4—C5	120.2 (2)	H10A—C10—H10C	109.00
C4—C5—C6	120.2 (2)	H10B—C10—H10C	109.00
C1—C6—C5	119.21 (17)	N2—C11—H11A	109.00
S1—C7—N1	123.50 (14)	N2—C11—H11B	109.00
S1—C7—N2	112.07 (12)	C12—C11—H11A	109.00
N1—C7—N2	124.42 (15)	C12—C11—H11B	109.00
N2—C8—C9	112.37 (18)	H11A—C11—H11B	108.00
N2—C8—C10	120.73 (16)	O1—C12—H12	109.00
C9—C8—C10	126.90 (18)	C11—C12—H12	109.00
S1—C9—C8	112.74 (15)	C13—C12—H12	109.00
N2—C11—C12	113.11 (15)	C12—C13—H13A	109.00
O1—C12—C11	111.54 (16)	C12—C13—H13B	109.00
O1—C12—C13	108.34 (16)	C12—C13—H13C	109.00
C11—C12—C13	109.20 (18)	H13A—C13—H13B	109.00
C1—C2—H2	120.00	H13A—C13—H13C	109.00
C3—C2—H2	120.00	H13B—C13—H13C	109.00
C2—C3—H3	120.00		
			
C9—S1—C7—N1	178.77 (15)	C8—N2—C7—N1	−178.65 (16)
C9—S1—C7—N2	−0.17 (14)	C8—N2—C11—C12	−94.2 (2)
C7—S1—C9—C8	0.00 (17)	N1—C1—C2—C3	−178.13 (16)
C7—N1—C1—C2	−127.80 (17)	C2—C1—C6—C5	0.7 (3)
C7—N1—C1—C6	54.5 (2)	N1—C1—C6—C5	178.37 (16)
C1—N1—C7—S1	3.0 (2)	C6—C1—C2—C3	−0.4 (3)
C1—N1—C7—N2	−178.18 (16)	C1—C2—C3—C4	−0.2 (3)
C8—N2—C7—S1	0.27 (19)	C2—C3—C4—C5	0.4 (3)
C11—N2—C7—S1	−173.96 (13)	C3—C4—C5—C6	−0.1 (3)
C11—N2—C7—N1	7.1 (3)	C4—C5—C6—C1	−0.4 (3)
C7—N2—C8—C9	−0.3 (2)	N2—C8—C9—S1	0.1 (2)
C11—N2—C8—C9	173.93 (17)	C10—C8—C9—S1	−178.99 (18)
C7—N2—C8—C10	178.92 (18)	N2—C11—C12—O1	52.9 (2)
C11—N2—C8—C10	−6.9 (3)	N2—C11—C12—C13	172.58 (15)
C7—N2—C11—C12	79.4 (2)		

### Hydrogen-bond geometry (Å, º)

**Table:**

*D*—H···*A*	*D*—H	H···*A*	*D*···*A*	*D*—H···*A*
O1—H1*A*···Cl1	0.82	2.36	3.1681 (16)	169
N1—H1···Cl1^i^	0.86	2.34	3.1675 (14)	163
C11—H11*A*···Cl1^i^	0.97	2.81	3.6440 (19)	144

Symmetry code: (i) *x*, −*y*+1/2, *z*+1/2.

## References

[bb1] Abdel-Wahab, B. F., Abdel-Aziz, H. A. & Ahmad, E. M. (2009). *Eur. J. Med. Chem.* **44**, 2632–2635. 10.1016/j.ejmech.2008.09.029 18995932

[bb2] Baia, M., Astilean, S. & Iliescu, T. (2008). *Raman and SERS Investigations of Pharmaceuticals*, pp. 125–142. Berlin, Heidelberg: Springer.

[bb3] Bruker (2005). *SADABS* Bruker AXS Inc., Madison, Wisconsin, USA.

[bb4] Bruker (2007). *APEX2* and S*AINT* Bruker AXS Inc., Madison, Wisconsin, USA.

[bb5] Farrugia, L. J. (1997). *J. Appl. Cryst.* **30**, 565.

[bb6] Farrugia, L. J. (1999). *J. Appl. Cryst.* **32**, 837–838.

[bb7] Lesyk, R., Vladzimirska, O., Holota, S., Zaprutko, L. & Gzella, A. (2007). *Eur. J. Med. Chem.* **42**, 641–648. 10.1016/j.ejmech.2006.12.006 17303290

[bb8] Liu, Z.-J., Fu, X.-K., Hu, Z.-K., Wu, X.-J. & Wu, L. (2011). *Acta Cryst.* E**67**, o1562. 10.1107/S1600536811019787 PMC3152027 21836975

[bb9] Lynch, D. E. & McClenaghan, I. (2004). *Acta Cryst.* C**60**, o815–o817. 10.1107/S0108270104023418 15528829

[bb10] Mohamed, S. K., Abdelhamid, A. A., Maharramov, A. M., Khalilov, A. N., Gurbanov, A. V. & Allahverdiyev, M. A. (2012*a*). *J. Chem. Pharm. Res.* **4**, 955–965.

[bb11] Mohamed, S. K., Abdelhamid, A. A., Maharramov, A. M., Khalilov, A. N., Nagiyev, F. N. & Allahverdiyev, M. A. (2012*b*). *J. Chem. Pharm. Res.* **4**, 966–971.

[bb12] Potikha, L. M., Turov, A. V. & Kovtunenko, V. A. (2008). *Chem. Heterocycl. Compd*, **44**, 86–91.

[bb13] Sheldrick, G. M. (2008). *Acta Cryst.* A**64**, 112–122. 10.1107/S0108767307043930 18156677

[bb14] Shiradkar, M., Kumar, G. V. S., Dasari, V., Tatikonda, S., Akula, K. C. & Shah, R. (2007). *Eur. J. Med. Chem.* **42**, 807–816. 10.1016/j.ejmech.2006.12.001 17239490

[bb15] Soliman, A. M., Mohamed, S. K., El Remail, M. A. & Abdel Ghany, H. (2012). *Eur. J. Med. Chem.* **47**, 138–142. 10.1016/j.ejmech.2011.10.034 22093758

[bb16] Spek, A. L. (2009). *Acta Cryst.* D**65**, 148–155. 10.1107/S090744490804362X PMC2631630 19171970

[bb17] Wang, M.-F. (2011). *Acta Cryst.* E**67**, o1581. 10.1107/S1600536811020873 PMC3151766 21836991

[bb18] Wu, Y. J. & Yang, B. V. (2007). *Prog. Heterocycl. Chem.* **18**, 247–275.

